# The endovascular treatment strategies of cerebrovascular injuries in traumatic brain injury

**DOI:** 10.1016/j.cjtee.2025.01.001

**Published:** 2025-01-22

**Authors:** Shuo Leng, Wentao Li, Yu Cai, Yi Zhang

**Affiliations:** a Center of Interventional Radiology & Vascular Surgery, Department of Radiology, Zhongda Hospital, Medical School, Southeast University, Nanjing, 210009, China; b Department of Radiology, Medical School, Southeast University, Nanjing, 210009, China; c Nurturing Center of Jiangsu Province for State Laboratory of AI Imaging & Interventional Radiology, Department of Radiology, Zhongda Hospital, Medical School, Southeast University, Nanjing, 210009, China

**Keywords:** Endovascular treatment, Traumatic cerebrovascular injury, Stroke, Vascular lesions

## Abstract

Vasculature injury occurs rarely in traumatic brain injury but increases lifetime risk of ischemic or hemorrhage stroke. The diverse and nonspecific clinical manifestations make the diagnosis and treatment of these injuries highly challenging. With advancements in device design, endovascular treatments have become widely adopted, playing an increasingly vital role in the management of vascular diseases. The purpose of this review is to introduce and summarize endovascular treatments of traumatic cerebrovascular injury and other related pathological states after traumatic brain injury. Given the innovations of neuroendovascular devices and improvements in the techniques over the past decade, this review will outline several recent advancements in endovascular treatment strategies for cerebrovascular pathologies. Popularizing more treatment options to clinicians will benefit in dealing with a variety of clinical scenarios and reduce the overall morbidity of traumatic cerebrovascular injury.

## 1 Introduction

Although global burden of injury diseases was decreasing in the last decade (2010–2021), trauma remains one of the leading causes of death worldwide.^1^ It is also the main cause of death among young people, especially those under the age of 45. Traumatic cerebrovascular injury (TCVI) is the predominant cause of death related to central nervous system trauma and is estimated to occur in 0.8%–1.8% of traumatic brain injury (TBI) cases.^2^ TCVI presents with a wide range of manifestations including different vessel injuries or pathological status like artery dissection, occlusion, spasm, venous sinus thrombosis, and carotid-cavernous fistula. However, TCVI does not present specific symptoms, which can vary depending on the severity and location of the injury and may be concealed by nearby neurological deficits. Undetected thus untreated TCVI poses a latent peril for those patients who have been injured.^3^

Noninvasive imaging, such as computed tomography angiography (CTA) and magnetic resonance angiography (MRA), is preferred for stable patients.^4^, ^5^, ^6^ However, in real clinical practice, emergency procedures or interventions are occasionally required to rescue patients experiencing ongoing ischemic or bleeding events. Moreover, interventional methods could treat the lesion as soon as diagnosed. Digital subtraction angiography (DSA) can rapidly diagnose the injured vasculature and treat it during the same procedure, providing both diagnostic and therapeutic benefits.

**Fig. 1:**
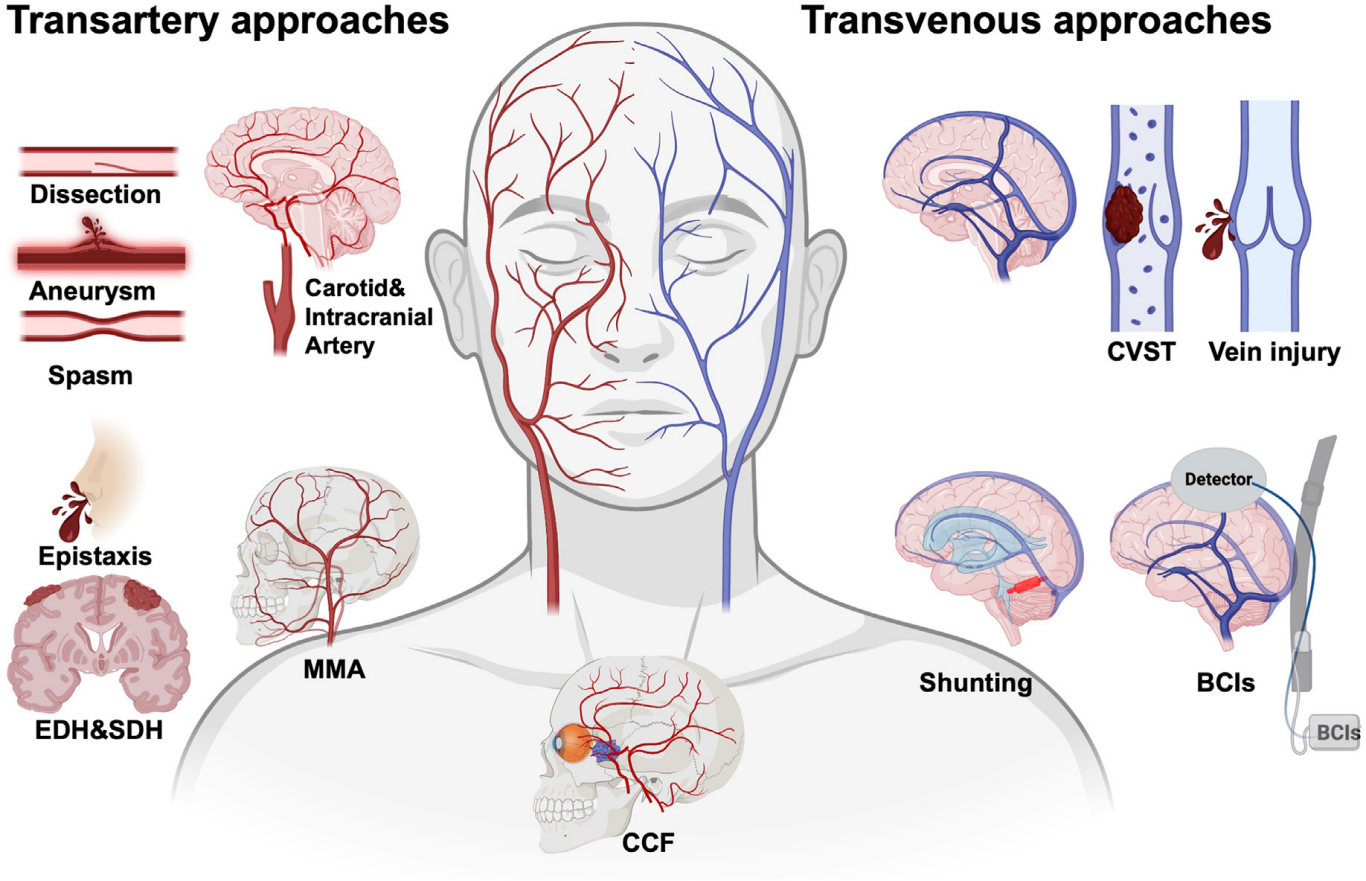
TCVI-induced pathological status and endovascular techniques for treatment of TCVI. TCVI: traumatic cerebrovascular injury; EDH: epidural hematoma; SDH: subdural hematoma; MMA: middle meningeal artery; CCF: carotid cavernous fistula; CVST: cerebral venous sinus thrombosis; BCIs: brain-computer interfaces. Created in BioRender. Shuo, L. (2024) BioRender.com/k47a270.

Historically, surgical and medical approaches have guided the management of TCVI, and endovascular techniques have emerged due to their minimally invasive nature. Endovascular treatment techniques, including transartery approaches and transvenous approaches, encompassing methods like stenting and embolization, could serve as definitive treatment in many traumatic instances and offer several advantages over traditional surgery.^3^^,^^7^^,^^8^ Fig. 1 shows that transartery approaches can be used to treat carotid and intracranial artery injuries like dissection, aneurysms, and spasm after subarachnoid hemorrhage (SAH), while transartery middle meningeal artery (MMA) embolization shows efficacy in epistaxis, epidural hematoma (EDH), and subdural hematoma (SDH). Cerebral venous sinus thrombosis (CVST) and vein injuries can be treated by transvenous approaches, while the eShunt system and brain-computer interfaces can also be placed by transvenous approaches. Carotid cavernous fistulas (CCFs) are the abnormal communications between the carotid artery and the cavernous sinus (CS), so they can be treated both transvenous and transarterial. These endovascular treatments include less procedural invasiveness, shorter recovery times, and the potential for better patient outcomes. This review will introduce endovascular treatment strategies for cerebrovascular pathologies. Offering more treatment options to clinicians will benefit them in determining the optimal treatment of TCVI.

## 2 Artery dissection

Artery dissection refers to a tear in the innermost layer of an artery and can present either with ischemia or hemorrhage.^9^ When an artery is dissected, blood can flow between the layers of the artery wall, causing it to bulge and easily rupture, and the compression of the normal luminal may lead to the occurrence of an ischemic event. According to the location and histological features, traumatic cerebral artery dissection (TCAD) can be broadly divided into cervical artery dissection and intracranial artery dissection. Intracranial arteries are defined as the intradural portion in which after C6 in the internal carotid artery and after V4 in the vertebral artery. An intracranial artery is also characterized by features of lack of elastic fibers in the media, little adventitial tissue, and no external elastic lamina.

The incidence of traumatic internal carotid artery dissection is up to 0.86% while the incidence is 0.53% for traumatic vertebral artery dissections, but the incidence of traumatic intracranial artery dissection is rarely reported.^10^ For non-traumatic artery dissection researches revealed that intracranial artery dissection (IAD) with a predilection for the Asian population and the posterior circulation.

TCAD usually presents with various symptoms and manifestations, such as headache, cervical pain, Horner syndrome, and cranial nerve palsy partly due to compression of adjacent structures. SAH exceptionally occurs in TCAD when the dissection expands to the intradural portion.

The diagnosis of TCAD is based on manifestations and imaging findings. For patients with suspected injuries around the neck, CTA is recommended for the initial screening test.^11^ Although CTA may be immediately performed as an initial screening when patients are administered, DSA is the golden standard for identifying a possible vasculature injury.^12^ Other imaging methods like duplex ultrasonography and MRI have their unique advantages. Duplex ultrasonography has the advantage of being a non-invasive method and can be performed bedside, but its reliability is strongly affected by the experience of the sonographer. MRI helps diagnose acute ischemia secondary to dissection. MRA could be an alternative for TCAD detection and has superiority in the identification of intramural hematoma over CTA. The vessel wall imaging using MRI permits the evaluation of the pathophysiology of the artery wall. Significantly but hardly detected pathognomonic features like intramural hematoma, intimal flap, and double lumen, all can be identified using magnetic resonance vessel wall imaging.^13^

There are no distinct guidelines regarding the proper diagnosis and therapeutic management of traumatic artery dissection but referring to the common characteristics between traumatic and spontaneous artery dissection, guidelines of spontaneous artery dissection still deserve reference. The appropriate treatment for TCAD remains debated, but the aim of the treatment of artery dissection is clear − to prevent those patients from stroke or recurrence of stroke.

### 2.1 Carotid artery dissection (CAD)

CAD is recognized as a leading cause of ischemic stroke (IS) in young people and accounts for 15%–25% of ISs in patients under the age of 45.^14^ IS usually occurs acutely after TBI, and patients with TBI have an increased risk of developing IS. Approximately 2% of patients who survived moderate-to-sever TBI were accompanied by cervical (carotid or vertebral artery) artery dissection, and CAD was identified as one of the highest risk factors of post-traumatic cerebral infarction.^15^

In patients with TCAD, treatment typically consists of antithrombotic therapy and endovascular repair. For antithrombotic therapy, the recently published guidelines do not make any differentiations between spontaneous and traumatic dissection, but the special issues should be taken into consideration. Antiplatelet and anticoagulation treatments are both recommended in patients with ischemic symptoms. Patients with radiographic high-risk features (e.g., severe stenosis or occlusion, intraluminal thrombus) and low risk of bleeding warrant anticoagulation therapy, while patients with a high risk of hemorrhage radiographic features (such as large infarct size, hemorrhagic transformation, and intradural extension of extracranial dissection) are more suitable for antiplatelet therapy. Studies have shown a decrease in neurologic sequelae with the use of antithrombotic or antiplatelet agents in these patients, with no significant difference between the 2 agents.^7^^,^^8^ Any choice of medical management was better than no treatment, but no specific differences between choice of medical management and stroke.^9^

For endovascular treatments, percutaneous transluminal angioplasty technique with a balloon catheter was rarely reported unless Walrus Balloon Guide Catheter^12^ was reported as a preliminary experience in treatment with a traumatic carotid dissection, while stenting is normally recommended as a secondary strategy for patients who refractory to maximal medical treatment. Although there have been reports of discouraging results (45% occlusion rate in the stents group compared with 5% in those who received antithrombotic agents alone) in the past study.^10^ Recent research^11^ showed a couraging result of 2.0% (1/50), and the authors believe that the lack of antiplatelet therapy may have played an important role in those poor outcomes. So, Galyfos et al.^11^ suggest the following criteria for stenting: (1) patients with recurrent symptoms despite medical therapy, (2) patients with hemodynamic hypoperfusion (involvement of multiple vessels or poor collateral vessels), (3) patients with expanding or symptomatic pseudoaneurysm, and (4) patients with contraindications to anticoagulation. There are various stents were reported to treat with TCAD no matter balloon-expandable^16^ or self-expandable, but balloon-expandable stents were used as an alternative option when other stent types unavailable in an emergent situation. A series of 6 patients with CAD were reported to introduce polytetrafluoroethylene-covered stent Hemobahn (W. L. Gore, Flagstaff, Ariz) endoprosthesis into the internal carotid artery (ICA) using hybrid open/endovascular technique and had a good outcome at 30-day follow up. The Enroute trans carotid neuroprotection system (Silk Road Medical; Sunnyvale, CA) was designed to combine the concepts of blood flow reversal to treat high-risk patients, and Safety and Efficacy Study for Reverse Flow Used During Carotid Artery Stenting Procedure^17^ study have shown a low stroke rate compared with other prospective trials of endovascular carotid intervention. Flow diverter stents which have proven to be an effective device for intracranial aneurysms with large necks also have satisfactory initial results in treating CAD.^18^^,^^19^ During the endoluminal procedure, it is most important to differentiate the true lumen from the false lumen and selective microcatheterization of the true lumen is challenging. Using super selective catheterization, angiography, and a microballoon catheter are reported effective in visualizing the true lumen. Intravascular ultrasonography and optical coherent tomography have been reported to be useful for the treatment of dissection at other locations, such as the coronary arteries and the aorta, its application to carotid artery dissection might also be feasible.^20^^,^^21^

### 2.2 Intracranial artery dissection

While CAD has been extensively studied, less data is available about IAD. The incidence of IAD is unknown but reported with a variety between different ethnic origins. The proportion of IAD among all cervico-cephalic dissection is 67%–78% in East Asia,^22^ which is much higher than in other populations. IAD more frequently affects the posterior circulation than anterior circulation, and the V4 segment is the most common site according to most series.

The 2 main clinical presentations of IAD are SAH and cerebral ischemia (ischemic stroke and transient ischemia attack).^22^ SAH occurs in 50%–60% of patients with IAD, while 30%–78% of IAD patients present cerebral ischemia. Headache is the most frequent symptom, and about 80% of patients have prodromal headaches. Other symptoms are caused by the mass effect, which affects the brainstem or cranial nerves mostly. The diagnostic imaging features are similar to CAD but more challenging due to the small size of the intracranial artery. Some studies reported that fusiform or irregular aneurysmal dilation of the intracranial artery was a more common feature in IAD patients with SAH than those without. Segmental stenosis and occlusion combined with SAH also suggest IAD.

Traumatic IAD is only a small percentage of all IAD, with a recent report of only 11.5%.^23^ For both medical and interventional (endovascular, surgery) management of traumatic intracranial artery dissection, no evidence-based guidelines^13^ have been published thus far. Unlike extracranial arteries, which have a structure of internal elastic lamina, elastic fibers in the media, and external elastic lamina, intracranial arteries are associated with a higher risk of bleeding.^6^ So, a more conservative antiplatelet treatment has been proposed-close monitoring instead of antiplatelet therapy. Surgical and endovascular treatment aims to reduce the blood flow into the dissected cavity and can be broadly divided into deconstructive techniques and reconstructive techniques. Deconstructive techniques sacrifice the parent artery, whereas reconstructive techniques aim to maintain the parent artery. Open surgical ligation of the dissected artery may be replaced with minimally invasive endovascular occlusion. Surgical treatment has also been reported for the reconstruction of the injured artery with in situ veins. However, the morbidity was high, with 58% experiencing cranial nerve injury and 10% suffering from a stroke in the largest reported series of patients with carotid dissection treated surgically.^24^ The Gateway dual-lumen angioplasty catheter was reported to perform percutaneous transluminal angioplasty in IAD and resulted in technical success.^25^ Reconstructive endovascular treatments, such as stents and flow diversion (FD), can be performed to separate the false and true lumens, illustrating successful off-label use without severe complications. Patients who received stents or FD implantation must adhere to antiplatelet therapy, which carries some risk of parenchymal hemorrhage, to prevent in-stent thrombosis. A balloon occlusion test should be preceded to evaluate the collateral supply before undertaking a deconstructive technique. Finally, emergent revascularization by mechanical or chemical thrombectomy can be considered to be performed in patients with acute large vascular occlusion due to artery dissection.

### 2.3 Traumatic aneurysms

Traumatic aneurysms are mostly pseudoaneurysms histologically and are found in less than 1%^26^ of all intracranial aneurysms. It is formed with products of the clotting cascade surrounding the ruptured injured artery, unlike the true aneurysm, which has a real artery wall. Clinical presentations are varied according to the location, size, and status of the aneurysms. More than half of the patients with pseudoaneurysms present with SAH, and headache is the most common symptom affecting more than 80% of patients.^27^ Clinical presentations like SAH, cervical bruit, LeFort II or III fracture, and basilar skull fracture, are also warning signs of traumatic aneurysms. If untreated, a high mortality rate of 50% has been reported for patients with intracranial pseudoaneurysms due to delayed rupture and disastrous bleeding.^28^

The “gold standard” diagnosis of aneurysms is also cerebral angiography, but due to the complex condition of patients at admission, the exact timing of when to perform the angiography is a matter of debate.^29^ Various studies have suggested that patients with the warning sign mentioned above should undergo a cerebral angiography as soon as possible. Delayed filling and stagnation of contrast agents are the characteristic DSA features of the pseudoaneurysm. Other features like the absence of the aneurysmal neck, an unusual site of occurrence, and an irregular contoured aneurysmal sac also contribute to the diagnosis.

The goal of treatment is to exclude the aneurysm from circulation to prevent its rupture. In the past, many reports demonstrated the efficiency of surgical repair. Surgical repair has been proven to be superior to surgical ligation or clipping, but later treatments can ensure a definitive exclusion of the aneurysm from circulation. Endovascular reconstructive surgery was as effective as deconstructive surgery to treat vertebral dissecting aneurysms was reported in a meta-analysis,^30^ and showed a lower periprocedural morbidity. Research about complete occlusion of the pseudoaneurysm with preservation of the parent vessel using coil embolization, stent-assisted coil embolization, stent angioplasty, covered stents placement or flow diverters also showed good results.^31^ Another deconstructive endovascular treatment of complete occlusion of the dissecting segment has been known as an optimal treatment, especially for vertebral artery dissections. In cases where therapeutic occlusion of the parent vessel is necessary, a balloon occlusion test can be performed under local anesthesia to assess collateral low both clinically and angiographically before therapeutic occlusion of the vessel. Clip anchor-assisted coil embolization is a hybrid surgery technique combined with endovascular occlusion and surgical clip of the parent artery and showed good results. Onyx™ (Medtronic, Minneapolis, MN, USA) was introduced as an alternative method when the coils cannot be placed in position, resulting in successful total occlusion.^32^

## 3 External carotid artery (ECA) injury

ECAs originate from the common carotid artery at the level of the superior border of the thyroid cartilage and supply the blood to the extracranial head and neck and most of the meninges intracranially. Injury to the ECA and its branches usually occurs following blunt or penetrating facial trauma and is variably reported in different clinical series (ranging from 1% to 11%).^33^, ^34^, ^35^ EDHs and SDHs are so-called extra-axial bleedings and are common clinical entities after TBI.^36^ The frequency of SDHs is 5 times that of EDHs in the case of TBI, and 3 times that in mild TBI, according to past reports. SDHs are more frequently seen in high-energy trauma, such as road accidents, and SDHs affect men more frequently than women according to literature.^36^ EDHs often have a favorable prognosis. Bir et al.^29^ reported a mortality rate of 3.6% in a large cohort, while SDHs have a wide-ranged perioperative mortality rate between 11.5% and 67.1%.

### 3.1 EDH

EDH located at the middle fossa usually originates from the tearing of the MMA.^37^ Surgery to evacuate the occupied effect of hematomas is the standard therapy. Although craniotomy is a mature surgical procedure that can provide complete evacuation of hematomas, it is a more invasive approach and brings general anesthesia risk. Burr hole drainage with or without injection of urokinase into the hematoma cyst was reported in recent years, which seems like could cover the shortage of the craniotomy. However, those therapies above mentioned can eliminate the occupied effect of hematomas but cannot prevent the injured MMA rebleeding.

Angiography is the gold standard for diagnosis of artery hemorrhage and some reports showed that combined with endovascular embolization could cease the bleeding point in EDH patients at the same time. Patients with small traumatic EDH who received MMA embolization could maintain the size stabilization and therefore avoid late hematoma expansion.^38^ MMA embolization combined with Burr hole drainage may be an effective and safe option for elder patients or those contraindicated for general anesthesia.^39^ The procedure of MMA embolization was easy to perform, and the materials used varied from temporal to permanent. Gelatin sponge (Gelfoam), 20% N-butyl-2-cyanocrylate (NBCA) (TruFill, Codman), polyvinyl alcohol particles, Onyx™ (Medtronic, Irvine, CA),^40^ platinum balls, or a combination was reported effectively. When dealing with the pseudoaneurysms of the MMA, which is a rare entity with a high risk of rupture without a complete artery wall, liquid agent Onyx™ may be a better choice.

To avoid complications, some considerations must be taken into during the procedure. There are some dangerous anastomoses between the ICA and ECA, so embolic agents larger than 100 μm in diameter and absorbable like Gelfoam may be a safe choice.

### 3.2 SDH

SDH is the most common type of intracranial bleeding and chronic subdural hematoma (cSDH) always occurs 3 weeks after the injury onset.^36^ Differently from acute subdural hematomas may be resolved completely, cSDH usually progresses.

Conservative management, such as frequent follow-up imaging, is often reserved for stable patients without significant brain compression or midline shift. Steroids and statins are drugs used to treat cSDH thought to inhibit the formation of the neomembrane and reduce inflammation at the endothelium. Results about steroids are conflicting, one systematic review found that patients treated with corticosteroids showed no therapeutic benefit, while another systematic review found that 83%–97% of patients returned to their neurological baseline.^41^^,^^42^ Statins may be considered more effective than steroids. Results showed that patients undergoing statin treatment have a lower chance of experiencing deteriorating mental status than those without.^43^

Although guidelines for surgical management of cSDH are lacking and burr hole drainage is a generally curative surgery way to treat cSDH, MMA embolization was reported as an effective and minimally invasive treatment in recent years. Burr hole drainage is effective in releasing the mass volume but with a recurrence rate reported up to almost 20%.^44^ MMA embolization was associated with a lower risk of recurrence compared with conventional management.^45^ And many previous studies reported MMA embolization may be effective in some cases of recurrent cSDH. cSDH more often occurs in elder patients, who have a higher risk under general anesthesia, and MMA embolization performed under local anesthesia may be more suitable.

Previous studies reviewed that MMA embolization has lower recurrence and comparable complication rates compared with conservative management. But insufficient data were available to compare differences in outcomes between burr holes *vs.* MMA embolization.^45^ Higher clinical evidence-level randomized control trials are underway to investigate the efficacy, safety, and utility of MMA embolization for cSDHs. Embolization materials were heterogeneous, including particles, liquid, coils, microspheres, and Onyx, all of which were reported to be effective, and the technique was successful. However, when embolizing the peripheral regions of the MMA, it may be better to use 20% NBCA mixed with lipiodol because of the low risk of catheter adhesion.^46^

Recent research found that the onset of migraines was associated with an increase in MMA circumference, so MMA was thought to be associated with chronic headaches and migraines.^47^ Thus some research focused on MMA embolization to treat headaches and found that patients with cSDH who underwent MMA embolization could improve the headache symptoms.^48^ Although MMA embolization is increasingly of interest, many questions need to be determined, including appropriate patient selection, efficacy as a stand-alone procedure, optimal embolization techniques and materials, and timing of embolization concerning surgical intervention in symptomatic patients.

### 3.3 Epistaxis

Epistaxis (nosebleed) is common in daily life, but epistaxis from a pseudoaneurysm is always needing immediate treatment.^49^ The incidence of post-traumatic epistaxis ranges from 1% to 11%, and the most involved vessel is the internal maxillary branch origin from ECA. Compressive therapy is the first step to controlling epistaxis. Nasal packing can be performed bedside and is effective in controlling mild to moderate bleeding; however, the effectiveness is reduced, and the recurrence rate is high with severe bleeding. Conservative management of epistaxis is generally successful, only approximately 6% of patients will require invasive management like transnasal ligation or endovascular embolization. Although arterial ligation is demonstrated more cost-effective, endovascular treatment still has favorable outcomes by being increasingly recommended as the primary treatment.^50^

Since 1974, endovascular embolization has emerged as an effective treatment for intractable epistaxis. Microcoils, NBCA, and Onyx were reported to be safe and effective in embolization, with a success rate of 75%–90%.^51^^,^^52^ A pre-embolization angiogram before an embolization is has been reported with a success rate of 75% in embolization, while important to identify the anastomoses between the ECA and ICA or ophthalmic artery that could increase the risk of stroke or blindness during embolization. And nonpermanent agent sized in the 200−700 μm would be the ideal particle in patients suspected of dangerous connections. Other techniques like detachable coils, detachable balloons, covered stents, and flow-diverting stents also had been reported successful.^53^

## 4 Cervical venous injuries

Injuries to both arteries and veins can result in bleeding, the latter of which is often overlooked.

### 4.1 Isolated vein injuries

Isolated vein injuries are rare and almost caused by penetrating trauma.^54^ Iatrogenic damage occurs commonly in central venous catheterization. The internal jugular vein courses superficially in the neck without the protection of bones or cartilage, making it susceptible to trauma. Internal jugular vein injuries represent 20% of all neck injuries.^55^

The optimal management is currently unknown. Although the injured vessel can be ligated with impunity in the majority of situations, the restoration of the outflow was given the highest priority.^56^ Nonoperative management, under careful advanced screening such as multislice helical CTA (M-CTA), is increasing in acceptance in patients with stable vital signs. Endovascular management has also been described as an alternative method when injuries occur near the jugular orifice with difficult surgical access.^57^^,^^58^ In these 2 cases, the lesions located at the jugular foramen made ligation impossible; detachable coils and NBCA were used to embolize the injured vein. Additionally, inserted foreign materials could increase the risk of infection, and broad-spectrum antibiotics must be taken into consideration to prevent infection.

### 4.2 CVST

CVST is a growing concerned complication of TBI. Direct mechanical damage of vascular endothelium and the blood flow deceleration caused by the hematomas overlying on the cerebral venous sinus are initial factors of thrombosis formation,^59^^,^^60^ and the hypercoagulable state caused by traumatic blood loss or dehydration therapy also contributes to thrombosis formation. The incidence of CVST had been reported to be up to 4%^61^^,^^62^ with penetrating trauma and extremely rare with blunt injury, but CVST has a high incidence of 23% in blunt trauma with a calvarial fracture crossing a dural venous sinus.

The presence of hemorrhagic infarcts in bilateral lobes, cord sign, “empty delta” sign, and the “dense vein” sign is indirect evidence of CVST. Unenhanced CT was usually the best noninvasive method for diagnosing CVT before, while MRI and magnetic resonance venography are more sensitive and specific for diagnosing CVST with reported sensitivities up to 95%. MRI vessel-wall imaging has shown excellent diagnostic value in acute CVST and can be used to predict treatment response to endovascular therapy.^63^

Anticoagulation treatment is the most common management of general CVST and gained the guideline recommendation of the European Stroke Organization in 2017. However, in the subtype of traumatic CVST, the risk of hemorrhage expansion must be taken into consideration. Results showed that commencing anticoagulation 48–72 h after the initial injury is associated with fewer deleterious effects. A 3- to 6-month duration is generally accepted as an appropriate treatment course.^64^^,^^65^ Patients who fail to improve or who have worsening neurologic deficits despite medical therapy may be considered for chemical or mechanical thrombolysis. Low-dose urokinase ^66^^,^^67^ was reported as a chemical thrombolysis option to treat patients with traumatic CVST and resulted in promising improvements in both functional and neuroradiological outcomes. Although endovascular thrombolysis is more aggressive, it should be reserved for those patients with heavy-burden thrombus. Endovascular thrombolysis could reduce hemorrhagic risk caused by the systemic dose.^47^ Endovascular strategies include intrasinus thrombolysis, balloon angioplasty, thrombectomy, and sinus stent placement.^68^^,^^69^ These techniques may be used in isolation or combination. One systematic review demonstrated a 69% radiographic resolution rate with mechanical thrombectomy in medically refractory cases, with a 14.3% mortality rate reported during the follow-up period.^70^ Additionally, recent reports have shown an improvement in intracranial pressure following mechanical thrombectomy and stent placement of dural sinuses.

## 5 Posttraumatic vasospasm (PTV)

Vasospasm refers to a sustained contraction condition of the intracranial artery, which can lead to cerebral ischemia or even infarction. Vasospasm can have a significant impact on functional outcomes after TBI,^63^^,^^71^ but is not routinely assessed unless clinical signs or symptoms suggest its presence, leading to underdiagnosis. Young patients, low GCS scores at admission, and more injured lobes are reported to be correlated with a high risk of developing PTV.^72^ Early angiographic studies reported PTV incidence rates between 5% and 19%;^73^^,^^74^ however, more recent neurosonography and imaging studies suggest a higher rate, ranging from 27% to 63% in adults and approximately 36% in the pediatric population.^73^, ^74^, ^75^ While the reported incidence of traumatic vasospasm ranges from 19% to 68%, the true incidence remains unknown due to variability in protocols for its detection.

In the absence of enough clinical data, treatment of PTV poses unique challenges. Nimodipine, which serves as a calcium channel blocker, is the most effective and widely used medication for the prevention of VSP after aSAH and is also reported to benefit patients with PTV.^76^ Although “3-H” (hypertension, hypervolemia, hemodilution) was previously used in managing vasospasm due to SAH as well as PTV, this management can potentially worsen cerebral edema and intracranial pressure if cerebral autoregulation is impaired.

Endovascular treatments represent an alternative strategy and typically involve intra-arterial vasodilators calcium channel-blocking medications (nicardipine and verapamil) or milrinone and/or balloon angioplasty. Balloon angioplasty is often performed for symptomatic vasospasm is effective in cases refractory to spasmolytic injection and is known to improve clinical outcomes. Recently, stent-retrievers and stent-like devices have been used to successfully treat cerebral vasospasm, pRELAX device;^77^ the Cascade device;^78^ Solitaire Stent Retriever^79^ were reported to be successful and effective. Scepter Mini balloon is a small-sized balloon catheter with dual lumens released by Microvention, which was reported as a technical experience in treating vasospasm after SAH.^80^ Percutaneous cervical sympathetic block has been reported as a not routinely used, but effective treatment for cerebral vasospasm.^81^

## 6 CCF

CCFs are the abnormal communications between the carotid artery or external carotid artery and the CS. They can be classified in different manners: CCFs are subdivided into traumatic or spontaneous according to the etiology; direct or indirect groups according to the fistula anatomically originated; high or low flow according to hemodynamics. Barrow classification based on the angiographic is the most widely accepted and guided treatment plan.^82^ According to the arterial supply of the fistula, CCFs are divided into 4 subtypes: subtype A is the fistula supplied directly by the ICA; subtype B, C, and D are supplied by the meningeal branches from ICA, ECA, and both, the subtypes B, C, D also called dural fistulas. So, subtype A is the direct CCF (dCCF) and subtypes B, C, and D are indirect CCF.

CCFs usually occur spontaneously or secondary to trauma, and trauma-associated CCFs are usually direct CCFs and are the majority of all the CCFs.^83^ The TBI with skull base fracture can puncture the cavernous segment of ICA courses through the CS resulting in the blood overflow from ICA to CS. The clinical presentations of direct CCFs can be various and progressive according to the venous pressure and arterial perfusion. After the drainage vein reaches a critical level, symptoms like pulsating exophthalmos and intracranial vascular murmur occur. If patients are without the integrity of the Wills-circle, the drainage vein will steal blood from the artery resulting in a cerebral ischemia condition and symptoms that correspond.

CCFs are a rare entity of trauma, and the presentations lack specificity, so it is difficult for clinicians without experience to diagnose. The imaging examinations can provide more useful information. CT can easily recognize bone fracture, tortuous and thickened ophthalmic veins, and the enlargement of the CS.^84^ MRI can supply similar findings to CT, but the best examination is cerebral DSA which can provide more information helping identify the location and the drainage way of the fistula. Once the construction of the fistula is clear, the treatment strategy is supposed to be designed.

ICA ligature or trapping used to be performed to treat CCFs before endovascular techniques were developed. These treatments have huge limitations: high risk of several sequelae, symptoms do not improve, low cure rate, and easy recurrence. With the development of endovascular techniques and new embolic materials like detachable balloon catheters, embellished coils, liquid embolic agents, covered stents, and recently FD stents, these shortages may be overcome. Direct CCFs are not self-limited diseases and patients with signs like visual impairment, progressive paresis of extraocular muscles, intractable orbital pain, bruit, and progressive exophthalmos are supposed to be treated emergently. Open surgery or radiosurgical^85^ treatments were also reported but still utilized adjuvant therapeutic options. Endovascular treatments are the first line of therapy and can be divided into transvenous, transarterial, transorbital, or a combination according to the access approach. Transarterial approaches were more common in the treatments of direct CCFs due to the usage of detachable balloons while transvenous and transorbital are more frequently practiced in indirect CCF. With the upgrading of the new embolic materials, the transvenous approach is more favorable if both transarterial and transvenous are feasible because of the high long-term success rate.^86^

Detachable balloons were widely used for more than 3 decades and were reported as a reliable agent that could achieve a high obliteration rate. However, approximately a quarter to 1/5 of patients with Type A CCF following detachable balloon treatment still require a complete ICA occlusion. Long-term follow-up researches^87^^,^^88^ shows a high rate of recurrence and pseudoaneurysm formation usually caused by premature balloon deflation and migration. Moreover, migrated balloons may block the ICA system and result in a catastrophic ischemic stroke. Coils were the most utilized in the embolization of CS and were associated with a slightly lower risk of overall complications.^89^ Besides, coils barely reflux to the ICA system from CS and are hardly able to be attached to the delivery microcatheter as liquid agents like Onyx. Coils embolization^90^ also reported the drawbacks of high cost, and mass effect, with balloon or stent assistance leading to complicated procedures and long radiation exposure. Kohta et al.^91^ reported the placement of coils in the middle-lateral part of the CS may compress the abducens nerve and there is a significant correlation (*p* = 0.04) between the coils volume and the abducens nerve palsy in the median follow-up time of 33 months. NBCA (TruFill; Codman and Shurtleff Inc, Raynham, Massachusetts, USA), and ethylene-vinyl alcohol copolymer (Onyx; eV3-Covedien, Irvine, California, USA) are 2 main liquid materials and Onyx are more common. Onyx is a polymer mixed with tantalum powder dissolved in dimethyl sulfoxide. It has been used alone or with the assistance of coils, and balloons for the treatment of dCCF with favorable results.^92^^,^^93^ This liquid material was introduced in the embolization of CS to reduce the number of coils and the compression of the CS wall. Every coin has 2 sides. Onyx as a liquid material has favorable features of slow precipitation and precise penetration. It has the disadvantages of a risk of regurgitation to the ICA system and entrapment of microcatheter which is rare but terrible. A covered stent can also be used in the treatment of dCCFs if the fistula site is clear. This technique could not only reconstruct and preserve the parent vessel but also simplify the procedure and shorten the operation time within 2 h. Unlike bare metal stents, covered stents are composed of fabric or graft materials, such as polytetrafluoroethylene covering the metal stent. The membrane of the stent could directly obliterate the fistula, and so do the perforators and branches. So, the placement of a covered stent must avoid the important arteries such as the ophthalmic artery, anterior choroidal artery, anterior spinal artery, posterior inferior cerebellar artery, and anatomical variations like fetal-type posterior communicating artery, and primitive trigeminal artery. The balloon-expandable model makes the covered stent stiff and difficult to deliver in the tortuous artery. The Wills covered stent (MicroPort, Shanghai, China) was the first specially designed covered stent for intracranial vascular diseases that may overcome these disadvantages and was approved by the Chinese Food and Drug Administration in 2013. The Willis stents designed with unique structure make it better delivery and precise positioning. FD devices were approved for treating intracranial aneurysms by the U.S. Food and Drug Administration in 2011. As a revolutionary technique, FDs have been applied to several “off-label” indications in the past few years.^94^ According to the hypothesis that flow and vessel remodeling over intraluminal prosthesis could exclude the aneurysms, FD may also be employed to exclude the flow from the fistula. Besides sporadic case reports, Wendl et al.^95^ were the first to report a case series of 14 patients who were treated dCCF with FDs. A total of 59 FDs were used, containing 24 Pipeline Embolization Devices (Medtronic, Irvine, California, USA) which were “off-label” usage, and 35 p64 Flow Modulation Devices (Phenox, Bochum, Germany) which indicates dCCF. The results showed FDs as a treatment for dCCF is cost-intensive, the number of FDs used is almost 4 on average. Although this treatment may improve the presentation of patients with good safety in recent research, long-term outcomes remain to be seen.^96^

Both transarterial and transvenous are proven techniques, and no matter whether detachable balloons, coils, liquid agents, or stent grafts are reliable materials, the treatment decisions are made according to the individual circumstance and the surgeons’ experiences.

## 7 New directions

A novel minimally invasive device, which was designed to treat communicating hydrocephalus, could deliver the cerebrospinal fluid through a shunt. This shunt could be positioned by an endovascular approach, thus overcoming the traditional ventriculoperitoneal shunt surgery shortcomings like multiple incisions (neck and abdominal), catheter traversing cortex, and white matter.^97^

**Fig. 2:**
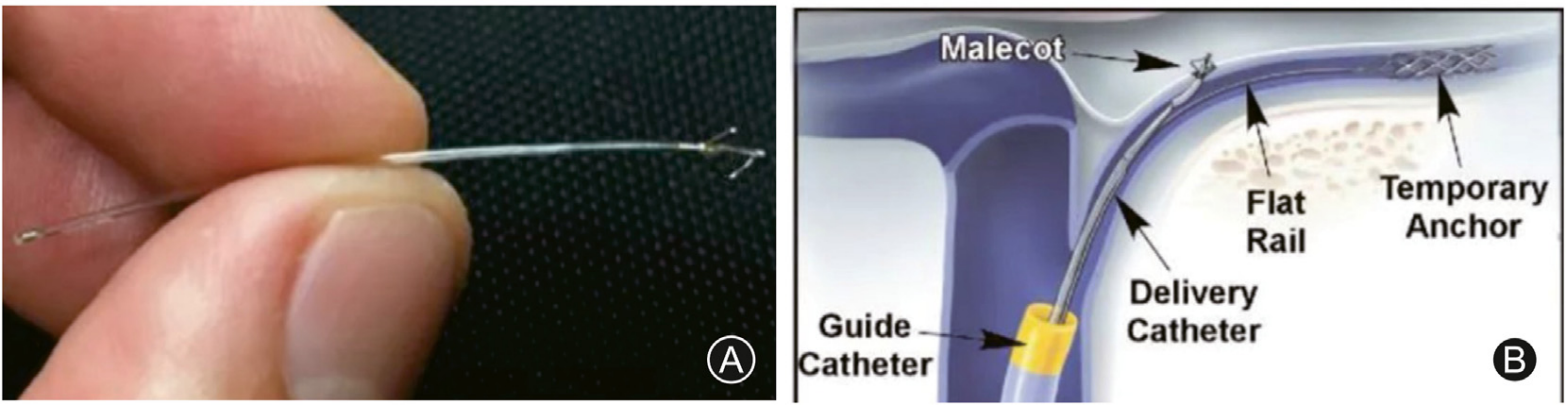
The eShunt system and diagram of the transvenous placement. (A) A close view of the eShunt system; (B) The malecot shaped anchor of the shunt was deployed in the cerebellopontine angle cistern and drained the cerebrospinal fluid into the internal jugular vein.^97^ Reproduced with permission from BMJ Publishing Group Ltd.

Post-traumatic hydrocephalus (PTH) is a kind of hydrocephalus that is commonly defined as a disorder of the production and absorption of cerebrospinal fluid in the ventricular system and occurs after TBI. PTH has been reported in 0.7%–29% of patients following TBI and resulted in a heavy burden on individuals and society.^98–98^ Mannitol and diuretics like Furosemide are dehydrating agents that can reduce intracranial pressure. Atorvastatin was reported to improve hydrocephalus and promote nervous system recovery.^99^ Surgical treatments include decompressive craniectomy and shunting, which include ventriculoperitoneal shunting, ventriculoatrial shunting, lumboperitoneal shunting, and endoscopic third ventriculostomy. Some previous studies have reported that 5%–23% of their TBI patients required shunt treatment due to PTH.^100^^,^^101^ And some patients who accepted shunt surgery early is associated with substantial clinical improvement.^101^ Patients with low-pressure hydrocephalus symptoms had the best shunt response,^102^ whereas patients with suspected vegetative state exhibited a minimal shunt response. However, patients may suffer from many complications including hemorrhage, infections, cerebrospinal fluid leak, shunt migration, and obstruction. A new transvenous trans-dural approach was developed to mitigate these disadvantages.^103^ The eShunt system (Fig. 2) is an implantable device that is equivalent to the arachnoid granulation physiologically, which was delivered via a transvenous transfemoral procedure and positioned in the cerebellopontine angle cistern. The endovascular approach succeeds in avoiding mechanical injury to traversed brain parenchyma and white matter, in contrast to conventional ventricular catheter-based surgical shunting.

Brain-computer interfaces (BCIs) are novel devices that can acquire signals from the brain, then analyze, translate, and deliver them to output devices that carry out desired actions. More and more disabled people are already using a BCI in their daily lives since a microelectrode array was implanted in a young man who completed tetraplegia after a C3-C4 cervical injury.^104^ Penetrating microelectrode arrays and subdural and epidural electrode arrays are approaches that require craniotomy to implant BCIs. Craniotomy and parenchymal damage would induce gliosis and scarring, resulting in the formation of a nonconductive barrier and attenuating neural signals. Endovascular electrode arrays could overcome this issue. Endovascular BCIs offer patients who are disabled after trauma an opportunity to have a more convenient lifestyle.^105^

The construction of trauma centers has become an innovative choice for many hospitals in recent decades.^106^ Interventional therapy is not only an important means of enhancing rescue capabilities in trauma center construction but also a key factor in promoting multidisciplinary collaboration, improving the quality of medical services, and reducing patient risks. With the advancement of technology and the deepening of practice, interventional therapy will continue to play an increasingly important role in trauma centers, thereby improving the prognosis and quality of life of trauma patients.

## 8 Conclusions

We summarized briefly the endovascular techniques for the treatment of TCV and TCVI-induced pathological status. Traumatic cerebrovascular injuries following TBI are common and pose a growing risk of both morbidity and mortality. Rapid detection and appropriate treatment are crucial for improving outcomes in patients with TCVI. While advancements in noninvasive imaging techniques like CTA and MRA have led to their increased use for screening, DSA remains the gold standard for diagnosing vascular injuries. Modern advances in neurointervention techniques and devices, along with emerging clinical evidence, offer opportunities to enhance the diagnosis and management of TCVI, thereby reducing the risk of significant long-term morbidity associated with improperly managed cerebrovascular trauma. At present, a global consensus has been gradually formed that endovascular treatment of head and neck trauma and vascular injury can save many patients' lives through "rapid bleeding control with embolization + vascular reconstruction", which is of great value. It is believed that endovascular treatment of TCVI will become one of the most important techniques in the whole process of trauma treatment in the future.

## CRediT authorship contribution statement

**Shuo Leng:** Writing – review & editing, Writing – original draft. **Wentao Li:** Writing – review & editing. **Yu Cai:** Data curation. **Yi Zhang:** Supervision.

## Ethical statement

Not applicable.

## Funding

The research was supported by the Jiangsu Province Science and Technology Support Project (No.: BE2023769)

## Declaration of competing interest

All authors declare no competing interest.
